# Microhabitat-specific restructuring of soil protist communities along a tropical land-use intensification gradient

**DOI:** 10.1093/ismeco/ycag132

**Published:** 2026-05-15

**Authors:** Gennuo Wang, Yan Zhang, Zheng Zhou, Carina C M Moura, Zuopeng Liu, Xu Xu, Siti Meliah, Christian Stiegler, Fabian Brambach, Martyna M Kotowska, Rahayu Widyastuti, Ingo Grass, Catrin Westphal, Stefan Scheu, Valentyna Krashevska

**Affiliations:** Johann Friedrich Blumenbach Institute of Zoology and Anthropology, University of Göttingen, 37073 Göttingen, Germany; Johann Friedrich Blumenbach Institute of Zoology and Anthropology, University of Göttingen, 37073 Göttingen, Germany; Department of Ecology of Tropical Agricultural Systems, University of Hohenheim, 70599 Stuttgart, Germany; Forest Genetics and Forest Tree Breeding, University of Göttingen, 37077 Göttingen, Germany; Johann Friedrich Blumenbach Institute of Zoology and Anthropology, University of Göttingen, 37073 Göttingen, Germany; Johann Friedrich Blumenbach Institute of Zoology and Anthropology, University of Göttingen, 37073 Göttingen, Germany; Jiangsu Collaborative Innovation Center of Solid Organic Wastes, Nanjing Agricultural University, 210095 Nanjing, Jiangsu, China; Department of Genomic and Applied Microbiology, University of Göttingen, 37077 Göttingen, Germany; Department of Bioclimatology, University of Göttingen, 37077 Göttingen, Germany; Department of Biodiversity, Macroecology and Biogeography, University of Göttingen, 37077 Göttingen, Germany; Department of Plant Ecology and Ecosystems Research, University of Göttingen, 37073 Göttingen, Germany; School of Natural Sciences, Macquarie University, Sydney, NSW 2109, Australia; Department of Soil Science and Land Resources, Bogor Institute of Agriculture, 16680 Bogor, Indonesia; Department of Ecology of Tropical Agricultural Systems, University of Hohenheim, 70599 Stuttgart, Germany; Agroecology & Functional Agrobiodiversity, Department of Crop Sciences, University of Göttingen, 37077 Göttingen, Germany; Centre of Biodiversity and Sustainable Land Use, University of Göttingen, 37077 Göttingen, Germany; Johann Friedrich Blumenbach Institute of Zoology and Anthropology, University of Göttingen, 37073 Göttingen, Germany; Centre of Biodiversity and Sustainable Land Use, University of Göttingen, 37077 Göttingen, Germany; Johann Friedrich Blumenbach Institute of Zoology and Anthropology, University of Göttingen, 37073 Göttingen, Germany; Senckenberg–Leibniz Institution for Biodiversity and Earth System Research, Biodiversity and Climate Research Centre, Functional Environmental Genomics, 60325 Frankfurt, Germany

**Keywords:** tropical ecosystems, litter, rhizosphere, environmental filtering, niche breadth

## Abstract

Microhabitat-specific responses of soil organisms to land-use intensification remain a major blind spot in biodiversity research. Here, we assessed how protists—key regulators of microbial diversity and nutrient cycling—differ in composition and roles across litter, rhizosphere, and bulk soil along a land-use gradient of increasing management intensity, from rainforest to shrubland, rubber plantations, and oil palm plantations in Sumatra, Indonesia. High-throughput sequencing revealed that rhizosphere protists responded most strongly to land-use intensification, with a 39.6% increase in Shannon index and marked shifts in community composition. Bulk soil protists showed similar but weaker responses, while litter protists exhibited compositional shifts without significant α-diversity changes. Notably, protist community composition was differentially structured by abiotic and biotic drivers across microhabitats independent of land-use type, with biotic dominance in the rhizosphere, abiotic dominance in litter, and joint control in bulk soil. To assess functional turnover, we applied an ecological niche framework (generalist–specialist–opportunist). Generalists remained stable in litter, whereas specialists showed reduced niche breadth and richness in rhizosphere and bulk soil, particularly in oil palm plantations, and opportunists showed intermediate responses. These findings demonstrate that land-use intensification restructures belowground communities in a microhabitat-specific and functionally predictable manner. By explicitly separating litter, rhizosphere, and bulk soil microhabitats, our study reveals microhabitat-specific assembly processes overlooked in conventional bulk-soil analyses and provides new insights into protist responses to land-use intensification in tropical soils. These findings highlight the need to incorporate microhabitat-scale processes when assessing soil biodiversity and ecosystem functioning under environmental change.

## Introduction

Soil is among the most biodiverse habitats on Earth, with a compact three-dimensional structure that promotes heterogeneity and supports life across trophic levels [1, 2]. Soils regulate biogeochemical cycles, store carbon, and sustain biodiversity [2, 3]. Different land-use systems (e.g. forest, cropland, and urban land) strongly alter soil biodiversity. Beyond differences among systems, land-use intensification (i.e. increased management inputs and disturbance within a given land-use type) further threatens key soil ecosystem functions, including nutrient cycling, soil biological productivity (i.e. microbial biomass production and metabolic activity), and the organization of belowground trophic interactions [4, 5]. Such land-use–driven transformations are particularly severe in tropical ecosystems, which host much of global biodiversity and carbon but face rapid degradation [6–8]. Conversion of rainforest to rubber and oil palm plantations degrades soils, disrupts nutrient cycles, and weakens microbial communities, reducing the resilience of belowground soil biota and associated processes [7, 8]. Indonesia, a hotspot of land-use change, provides a unique setting to study belowground biodiversity responses [9, 10].

Land-use intensification affects soil microbial systems by altering the two principal carbon pathways—litter-derived and root-derived inputs—that sustain soil food webs [11, 12]. The litter layer links above- and belowground systems through plant litter input and is structured mainly by abiotic factors—moisture, temperature, and organic matter—regulating microbial activity and decomposition [13, 14]. By contrast, the rhizosphere is a biologically enriched zone where root exudates—sugars, amino acids, and secondary metabolites—stimulate microbial growth and shape interactions among microbes [11, 15]. Bulk soil is shaped mainly by longer-term soil properties such as pH and nutrient availability [16, 17]. Soil compartments (i.e. litter, rhizosphere, and bulk soil, hereafter referred to as distinct soil microhabitats) act as distinct ecological filters shaping microbial communities and may, in some contexts, exert stronger effects than host plant identity or soil type [17–19]. Their functions vary across ecosystems, potentially altering community assembly. For instance, in oil palm plantations the litter layer primarily acts as a moisture-retaining layer, while its rapid decomposition limits its contribution to nutrient cycling and alters microbial communities [20]. Land-use intensification further reduces litter input, changes root exudates, and alters soil structure, reshaping microbial dynamics and carbon fluxes [21, 22]. These results highlight regional variability in litter and rhizosphere dynamics for predicting microbial responses and ecosystem stability under land-use change.

The response of bacteria and fungi to land-use change is well studied [5, 22], but protists—unicellular eukaryotes dominating microbial eukaryotic diversity—remain underexplored [10, 23, 24]. They regulate bacterial and fungal populations, enhance nutrient mineralization, and serve as prey for higher trophic organisms such as nematodes [24, 25]. Rainforest conversion alters protist community composition and trophic structure, highlighting their sensitivity to ecosystem change [10, 26]. Recent studies show that land-use intensification in croplands may increase local diversity (α-diversity) while reducing differences among sites (β-diversity), leading to more homogeneous communities [4, 27]. Although protist responses to climate, soil properties, and vegetation have been widely examined [28, 29], the mechanisms driving their responses to land-use intensification remain poorly understood, particularly in tropical ecosystems. This knowledge gap may be even greater at finer spatial scales, where soil microhabitats with contrasting abiotic and biotic conditions act as ecological filters shaping protist community assembly [17, 30]. However, most previous studies have characterized soil protist communities using bulk soil samples, implicitly assuming environmental homogeneity and potentially overlooking strong heterogeneity among litter, rhizosphere, and bulk soil. Land-use intensification can modify these microhabitat conditions by homogenizing soils and altering trophic interactions [4, 27, 31], underscoring the need to clarify protist assembly mechanisms under land-use change.

The way species respond to environmental change depends on niche breadth, defined as the range of conditions and resources they can exploit [32, 33]. Generalists with broad niches persist across diverse conditions and are more resilient, whereas specialists with narrow niches are adapted to stable, resource-specific environments and are more disturbance-prone [34–36]. Opportunists occupy intermediate niches, exploiting transient resources but often failing under long-term change [37, 38]. Shifts in the generalist–specialist ratio reflects land-use intensification, favouring generalists and leading to homogenization and functional loss [4, 27, 37]. This framework is relevant for protists, which are highly responsive to change and serve as indicators of soil functioning [23, 39, 40]. Understanding their niche strategies across microhabitats is key to evaluating biodiversity and resilience under land-use intensification.

Here, we investigated how conversion of rainforest to shrubland, rubber plantations, and oil palm plantations, together with distinct soil microhabitats (litter, rhizosphere, and bulk soil) structures protist diversity and community composition in Sumatra in relation to biotic and abiotic drivers, and tested the following hypotheses: (1) Land-use intensification alters protist diversity and community composition, with stronger effects in the litter layer, followed by rhizosphere and bulk soil. (2) The relative importance of environmental factors differ among microhabitats: abiotic factors dominate in litter, biotic interactions in the rhizosphere, and both in bulk soil. (3) Protists exhibit strategy-specific responses to land-use intensification: generalists maintain broad niches and stable distributions across microhabitats, with specialists showing niche contraction and opportunists displaying intermediate sensitivity.

## Materials and methods

### Study area and sampling

This study, conducted in Jambi, Sumatra, as part of the CRC990/EFForTS project [41, 42], compared rainforest, shrubland, rubber plantations, and oil palm plantations along a land-use intensification gradient, defined by increasing management inputs and disturbance (vegetation simplification, agrochemical inputs, and harvesting frequency) [7, 41, 43]. Along this gradient, rainforest represents the least intensively managed system, followed by shrubland and rubber plantations, whereas oil palm plantations represent the most intensively managed system, characterized by high fertilizer input and frequent use of agrochemicals [41, 44, 45]. Site details are provided elsewhere [42, 46, 47]. Across four land-use systems, a total of 126 circular plots (1000 m^2^ each) were established, each comprising five subplots (25 m^2^), distributed among rainforest (*n* = 33), shrubland (*n* = 30), rubber plantations (*n* = 30), and oil palm plantations (*n* = 33) (Supplementary Figs. 1–2).

Samples were collected between May 2021 and August 2022 using a cylindrical steel corer (5 cm in diameter) to a depth of 10 cm. Five cores were taken per plot. From each core, litter was carefully separated from the underlying soil using a clean knife and placed in labelled zip-lock bags (350 × 450 mm), while the soil was placed in a second labelled bag. All samples were transported on ice to the field facility and processed immediately. In the laboratory, samples were separated into three compartments: litter, rhizosphere (fine roots with minor amounts of adhering soil), and bulk soil. Litter (50–400 g fresh weight per plot) was homogenized, and 10 g was taken for lyophilization. Soil (~1 kg per plot) was sieved (0.5 cm and 1 mm mesh) to separate roots, and 15 g of bulk soil was taken for lyophilization. Fine roots (<2 mm diameter) with adhering soil were carefully separated manually, blotted dry, weighed, and stored on silica gel before lyophilization. To minimize contamination, gloves were worn and tools cleaned with water and 70% ethanol between samples. All samples were frozen at −80°C overnight and subsequently lyophilized using a VirTis Bench Top K lyophilizer (SP Industries, Warminster, PA, USA) at the University of Jambi (Indonesia). After freeze-drying, aliquots (~250 mg soil, ~150 mg litter, and ~80–100 mg roots) were used for DNA extraction: soil and litter using the DNeasy PowerSoil Kit (Qiagen, Germany) and roots using the InnuPREP Plant Kit (Analytik Jena AG, Germany).

### DNA extraction and amplification

DNA was extracted following the manufacturer’s instructions. The hypervariable V4 region of the 18S rRNA gene was amplified using the general eukaryotic primers TAReuk454FWD1 (5′-CCAGCASCYGCGGTAATTCC-3′) and TAReukREV3 (5′-ACTTTCGTTCTTGATYRA-3′) [48], with MiSeq-specific adapter overhangs added to both primers. The TAReuk454FWD1/REV3 may introduce taxon-specific amplification biases, with reduced representation reported for certain protist lineages such as Rhizaria, Excavata, and Apicomplexa. Therefore, relative abundances should be interpreted as patterns within the detectable community rather than absolute estimates of taxonomic composition [23, 48]. Polymerase chain reaction (PCR) amplifications were performed in 50 μl reactions containing 10 μl of 5× Phusion GC Buffer, 1 μl each of forward and reverse primers (10 μM), 1 μl MgCl₂ (50 mM), 1 μl dNTPs (10 mM), 2.5 μl DMSO, 0.5 μl Phusion High-Fidelity Polymerase (1 U; Thermo Fisher Scientific, Germany), and 1 μl of template DNA. Thermocycling conditions included an initial denaturation at 98°C for 1 min, followed by 35 cycles of 98°C for 30 s, 60°C for 45 s and 72°C for 1 min, and a final extension at 72°C for 5 min. The expected amplicon length was ~400 bp. To ensure reproducibility, all PCR reactions were performed in triplicate, pooled in equimolar concentrations and purified. Library quantification was performed using a Qubit fluorometer. Sequencing was performed on an Illumina MiSeq platform at the University of Göttingen Genomics Laboratory, following the protocol as described in a previous study [10] generating paired-end reads.

### Bioinformatics analysis

Sequence analysis used NGS-4-ECOPROD, a metabarcoding pipeline for 16S/18S rRNA gene data developed at the University of Göttingen, including quality filtering, amplicon sequence variant (ASV) inference, and taxonomic assignment (https://github.com/dschnei1/ngs4ecoprod).

Paired-end sequencing data from Illumina MiSeq were processed using a quality control and ASV clustering pipeline. Reads were quality-filtered using fastp (v0.23.4) [49], with default settings except for an increased per-based Phred score threshold of 20, base pair corrections via overlap (-c), and 5′- and 3′-end trimming using a sliding window of 4, a mean quality score of 20, and a minimum sequence length of 250 bp. Merged paired-end reads were processed using VSEARCH (v2.27.0) [50], with primer removal performed via cutadapt (v4.7) [50]. Merged amplicons were filtered to ≥350 bp and dereplicated using VSEARCH (--derep_fulllength). ASVs were identified using UNOISE3 (--cluster_unoise --minsize 8), followed by *de novo* (-uchime3_denovo) and reference-based (-uchime_ref) chimera removal against the SILVA SSU-NR database (v138.1). Taxonomic assignments were performed using BLAST (v2.15.0+) [51] against the Protist Ribosomal Reference Database (PR2, v5.0) [52].

After quality filtering, 10 260 eukaryotic sequences were retained. Singleton and doubleton ASVs were removed. In addition, all ASVs assigned to non-protist eukaryotes, including Metazoa, Fungi, Rhodophyta, and Embryophyta, as well as other multicellular or photosynthetic lineages, were excluded. After filtering, 3616 protist ASVs remained for downstream analyses. Taxonomic assignments were further validated using established frameworks [29, 53, 54]. Six low-depth samples (<500 reads) were excluded, leaving 372 samples for diversity analyses (litter, *n* = 124; rhizosphere soil, *n* = 125; bulk soil, *n* = 123).

### Statistical analysis

All analyses and visualizations were performed in R 4.4.0 (R Core Team, 2024). All data and analysis scripts are available on Zenodo (DOI: 10.5281/zenodo.18566804).

Community diversity and composition were analysed using *phyloseq* [55] and *vegan* [56]. Alpha-diversity indices were computed using the estimate_richness^()^ function, and beta-diversity was assessed using Bray–Curtis dissimilarities, principal coordinates analysis (PCoA), and permutational multivariate analysis of variance (PERMANOVA), with multivariate dispersion evaluated using Permutational Analysis of Multivariate Dispersions (PERMDISP), all implemented in the *vegan* package with 999 permutations. Linear models (LMs) were fitted using the base *stats* package, while linear mixed models (LMMs) were fitted using *nlme* [57] and *lme4* [58] package. Factor significance was evaluated using analysis of variance (ANOVA) in R, followed by Tukey’s HSD tests (*agricolae* package) [59]. Spatial autocorrelation (Moran’s *I*) was tested using *spdep* [60] with a K-nearest neighbour spatial weight matrix. Random forest regression was performed using the *randomForest* package [61]. Spearman correlation matrices were calculated using the corr.test^()^ function from the *psych* package and visualized using *corrplot*. Niche breadth was calculated using Levins’ index [62], and generalist–specialist classification was refined based on Shannon entropy. Structural equation modelling (SEM) was conducted using the *piecewiseSEM* framework [63]. Data visualization was conducted with *ggplot2* [64], and final figures were edited in Adobe Illustrator 2021.

#### Model selection for assessing land-use and microhabitat effects on protists

To assess the effects of land-use change and its interaction with microhabitat on protist communities, we compared LMs and LMMs using the Akaike Information Criterion (AIC). LMMs including ‘Region’ as a random intercept did not improve model fit and were not retained. LMs showed that the interaction model (land-use system × microhabitat; AIC = 677.40) outperformed the land-use system-only (AIC = 782.38) and microhabitat-only (AIC = 717.44) models. ANOVA confirmed that land-use system, microhabitat, and their interaction significantly affected protist Shannon index (all *P* < .001; Supplementary Tables 1–2). Spatial autocorrelation of LMM residuals was assessed using Moran’s *I* statistic [65, 66], based on a K-nearest neighbour spatial weight matrix constructed from sampling coordinates. No significant spatial clustering was detected (Moran’s *I*, *P* = .43), indicating no spatial bias in the residuals (Supplementary Table 3).

#### Analysis of protist α- and β-diversity

We assessed protist α-diversity using the Shannon index. Analyses were conducted across microhabitats and land-use systems. Differences in α-diversity were assessed using Tukey’s HSD test with a significance threshold of *P* <.05. As rarefaction did not alter diversity patterns, rarefied data were used for all main analyses, with non-rarefied results provided for comparison (Supplementary Fig. 3; Supplementary Table 4).

For β-diversity analysis, data were normalized using the Hellinger transformation [65] and Bray–Curtis dissimilarities were used for PCoA and PERMANOVA [66, 67]. Pairwise group comparisons were performed with pairwise.adonis^()^ with significance set at *P* <.05. To test whether land-use intensification caused true community homogenization (i.e. changes in dispersion rather than only centroid shifts), multivariate dispersion was analysed using *betadisper* with PERMDISP, conducted separately for each microhabitat.

#### Analysis of biotic and abiotic factors

Processed abundance data for bacteria, fungi, and nematodes were used for downstream analyses. We investigated abiotic (air temperature, canopy openness, carbon, nitrogen, water content (H_2_O), and pH) and biotic (tree richness, shoot biomass, root biomass, and bacteria, fungi, and nematode diversity) factors of protist diversity and community composition. Collinearity among variables was assessed using variance inflation factors (VIFs) and pairwise Pearson correlations (Supplementary Fig. 4), with highly collinear variables (VIF > 5 or *r* > 0.7) iteratively removed. Environmental data sources are detailed in Supplementary Table 5–6.

To identify the relative importance of environmental drivers for protist α-diversity (Shannon index), we applied a Random Forest analysis [61]. Model performance was evaluated using the out-of-bag *R*^2^. Variable importance was assessed using the %IncMSE metric, which quantifies the increase in prediction error when each predictor is permuted. Complementary univariate linear regressions provided *R*^2^ and *P* values for each predictor. Associations between protist β-diversity (based on Bray–Curtis PCoA1 scores) and environmental factors were tested with Mantel tests. Pairwise associations among biotic and abiotic factors were assessed using Spearman correlation coefficients (R, corr.test^()^, *psych* package).

To assess direct and indirect effects on protist communities, we applied piecewise structural equation modelling (piecewise SEM) based on *a priori* hypotheses describing the relationships among land use, microclimate, plant, soil, and biotic variables. Land use was represented by tree richness, declining from rainforest to oil palm. Predictors included microclimatic factors (air temperature, canopy openness), plant factors (shoot, root biomass), microbial diversity (bacteria, fungi), animal diversity (nematodes), and soil abiotic factors (carbon, nitrogen, pH, water). All predictors were standardized using *z*-score transformation before model fitting to ensure comparability among variables**.** Sampling site was initially tested as a random effect but excluded due to negligible influence. Composite predictors were weighted by regression coefficients from their respective component models, and model fit was evaluated using Fisher’s *C* statistic in the *piecewiseSEM* framework (*P* > .05), as recommended for this approach [68, 69].

#### Analysis of diversity, niche breadth, and ecological classification

Diversity estimates were refined using residuals from linear regressions of log-transformed ASV richness against sequencing depth [70]. Levins’ niche breadth index was calculated for each ASV from its relative abundance across sampling sites, and group-level values for each protist functional group (generalists, specialists, and opportunists) were obtained by averaging across ASVs, with higher values indicating broader niches. To control for richness differences among protist groups, we applied a multi-diversity approach [27], in which the richness of each functional group was scaled to the mean richness across all samples for equal weighting. Niche breadth classification followed earlier work [71], linking broader distributions to generalists and narrower ones to specialists.

Generalists, opportunists, and specialists were classified following established approaches [37, 72]. Each treatment (4 land-use systems × 3 microhabitats) was considered a unique environment, resulting in 12 environments. Observed ASVs were counted per environment and 50 000 random permutations of the ASV–environment association matrix were performed, preserving ASV counts per environment. *Z*-scores and *P* values compared observed distributions with random expectations. Broadly distributed ASVs (*Z*-score > 0 or *P* < .1) were classified as generalists; those restricted to one or two environments (*P* < .05) as specialists and others as opportunists. Generalists occupy a wide range of conditions, while specialists adapt to specific microhabitats [37].

To validate ecological strategy classifications, Shannon entropy was incorporated as a complementary metric [73, 74], capturing richness and evenness. Higher values indicated generalists, lower values specialists, and thresholds were set at ≤0.30 for specialists, >1.30 for generalists, and intermediate values for opportunists. Threshold robustness was assessed using sensitivity analyses with ±0.1-unit variation, resulting in 2.5%–10.6% of ASVs changing category. Classifications from entropy and the null model were compared; consistent results were retained, while discrepancies were resolved in favour of entropy due to its abundance-weighted robustness. Overall, 62.4% of ASVs showed consistent classifications between methods, whereas 37.6% differed (Supplementary Tables 7–9). Final assignments are provided in Supplementary Table 10.

## Results

### Protist α- and β-diversity across microhabitats and land-use systems

**Figure 1:**
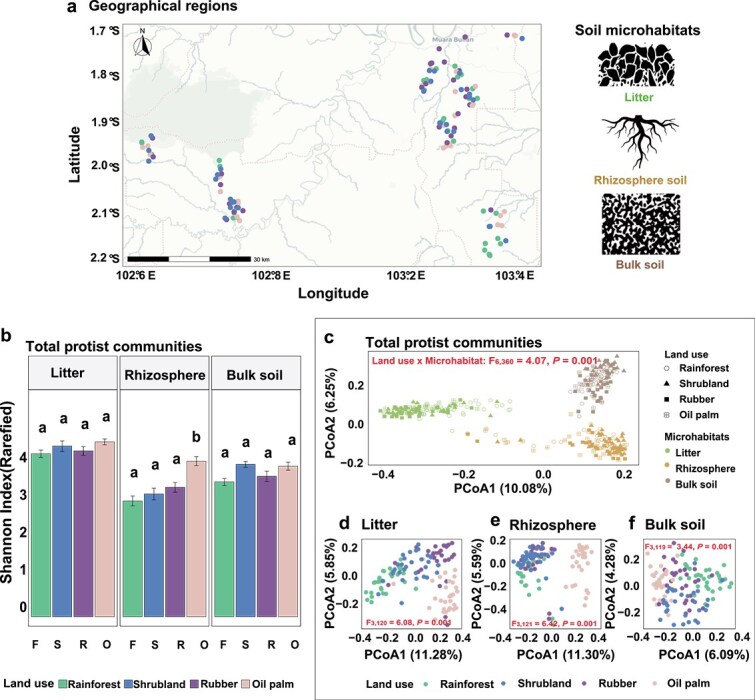
Protist α- and β-diversity across land-use systems and microhabitats. (**a**) Sampling locations with three soil microhabitats per site. (**b**) Shannon index of protist communities in litter, rhizosphere, and bulk soil under four land-use types. Bars represent mean ± SE; letters represent significant differences (ANOVA, Tukey’s HSD, *P* < .05). (**c**–**f**) PCoA of Bray–Curtis dissimilarities. (**c**) Combined, (**d**–**f**) by microhabitat. See Supplementary Table 12 for pairwise comparisons.

Protist α-diversity differed significantly between microhabitats (*F*_2,334_ = 57.71, *P* < .001) and land-use systems (*F*_3,334_ = 11.75, *P* < .001), but the responses depended on each other (significant land-use system × microhabitat interaction; *F*_6,334_ = 4.06, *P* < .001). Shannon index increased from rainforest to oil palm only in the rhizosphere (+39.6%; Fig. 1b; Supplementary Table 11), whereas changes in bulk soil and litter were not significant. Litter consistently supported higher diversity than rhizosphere and bulk soil but varied little among land-use systems. Observed ASV richness increased significantly with land-use intensification in the rhizosphere, but showed weaker responses in bulk soil and no significant differences in litter (Supplementary Fig. 5).

Protist community composition varied significantly across microhabitats and land-use systems as revealed by PCoA based on Bray–Curtis dissimilarities. Taxonomic composition of the 20 most abundant lineages is further illustrated in Supplementary Figs. 6–7. PERMANOVA confirmed significant main effects of land-use system (*R*^2^ = 0.05, *P* = .001), microhabitat (*R*^2^ = 0.15, *P* = .001) and their interaction (*R*^2^ = 0.05, *P* = .001) (Fig. 1c). Pairwise PERMANOVA tests indicated significant land-use effects within each microhabitat (Supplementary Table 12). In litter, rainforest communities were most distinct from oil palm, with shrubland and rubber intermediate (Fig. 1d). In rhizosphere, oil palm separated clearly from the other systems, which differed little among themselves (Fig. 1e). In bulk soil, separation was weaker with more overlap, but rainforest and oil palm remained most distinct (Fig. 1F). PERMDISP analyses further revealed that reduced β-diversity reflected true community homogenization only in the rhizosphere and bulk soil, but not in litter communities (Supplementary Fig. 8).

### Drivers of protist α- and β-diversity across microhabitats and land-use systems

**Figure 2:**
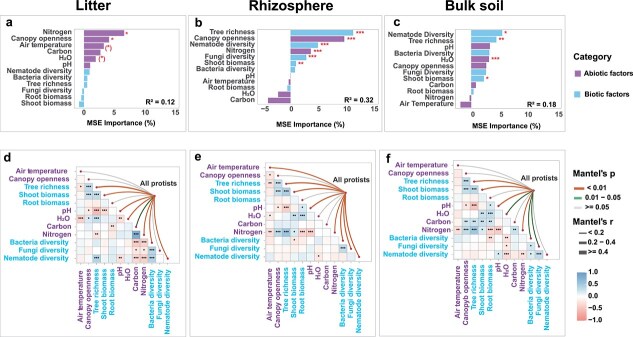
The influence of abiotic and biotic factors on α- and β-diversity of protist communities in litter, rhizosphere, and bulk soil. (**a**–**c**) Relative importance of abiotic and biotic predictors for α-diversity from Random Forest models. The *x*-axis shows mean squared error (MSE) importance (%). (**d**–**f**) Correlation networks of environmental variables with β-diversity. Box size indicates strength of Spearman’s correlation; edge thickness indicates Mantel’s r, while significance levels are shown in the legend (999 permutations). *R*^2^ denotes the out-of-bag (OOB) coefficient of determination of the Random Forest model. Significance: ^*^P < .05, ^**^P < .01, ^***^P < .001; ns = not significant.

Random Forest models revealed that the relative importance of biotic and abiotic factors of protist α-diversity varied across microhabitats (Supplementary Table 13). In litter, abiotic factors dominated, with nitrogen content and canopy openness strongest, followed by air temperature, carbon, and H_2_O (Fig. 2a). In the rhizosphere, biotic factors prevailed, with tree richness, nematode and fungal diversity, and shoot biomass as key drivers, while nitrogen and canopy openness were the only relevant abiotic contributors; this model had the highest explanatory power (*R*^2^ = 0.32) (Fig. 2b). In bulk soil, nematode diversity was most influential, followed by tree richness, H_2_O, and shoot biomass, with other abiotic variables playing only minor roles (Fig. 2c).

Mantel tests indicated that the strength of the association between environmental variables and protist β-diversity differed among microhabitats (Supplementary Table 14). In the litter, β-diversity correlated significantly with nitrogen, tree richness, nematode diversity, pH, and H_2_O (all *P* < .01) (Fig. 2d). In the rhizosphere, tree richness and nitrogen content showed the strongest associations, along with fungal and nematode diversity (all *P* < .01) (Fig. 2e). In the bulk soil, β-diversity was significantly correlated with tree richness, shoot biomass, pH, H_2_O, and nitrogen content, while bacterial and fungal diversity also showed significant but weak Mantel correlations (Fig. 2f).

### Structural pathways affecting protist α- and β-diversity across microhabitats

**Figure 3:**
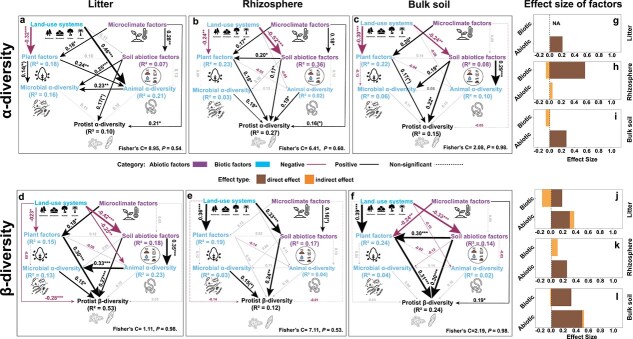
Structural equation models on the direct and indirect effects of abiotic and biotic factors. The α-diversity (upper panel) and β-diversity (lower panel) of protist communities in litter (**a**, **d**), rhizosphere (**b**, **e**), and bulk soil (**c**, **f**). Numbers on arrows represent path coefficients, with arrow widths proportional to their values. Arrow signs and labels indicate positive and negative relationships, while dashed arrows denote non-significant relationships. The *R*^2^ values represent the proportion of variance explained; ^*^P < .05, ^**^P < .01, and ^***^P < .001. Panels **g**–**l** show the effect sizes of biotic and abiotic factors on protist α- and β-diversity across the three soil compartments, displaying only significant effects; ‘NA’ indicates no significant relationship. See Materials and methods for details on the classification criteria of biotic and abiotic factors.

SEMs revealed contrasting drivers of protist α-diversity across microhabitats. In litter, diversity was mainly shaped by abiotic factors, with significant effects of soil and microclimatic variables, while biotic factors were not significant (Fig. 3a, g). In the rhizosphere, biotic interactions dominated, with strong contributions from microbial and animal diversity, alongside additional microclimatic and abiotic effects (Fig. 3b, h). In bulk soil, soil abiotic factors and plant factors were the main predictors (Fig. 3c, i).

Protist β-diversity showed similar microhabitat-specific drivers. In litter, it was shaped by soil abiotic factors, plant factors, microbial diversity, and negatively by land-use intensity (Fig. 3d, j). In the rhizosphere, microbial and soil abiotic factors dominated (Fig. 3e, k). In bulk soil, β-diversity was mainly explained by microclimatic, plant, and soil abiotic factors (Fig. 3f, l). Standard goodness-of-fit metrics for piecewise SEMs (Fisher’s *C* and AIC) are provided in Supplementary Table 15.

These effects likely reflect carbon input differences: rainforest had more litter but less root biomass, while oil palm showed the reverse (Supplementary Fig. 9), indicating contrasting carbon allocation.

### Protist niche breadth and diversity across microhabitats and land-use systems

**Figure 4:**
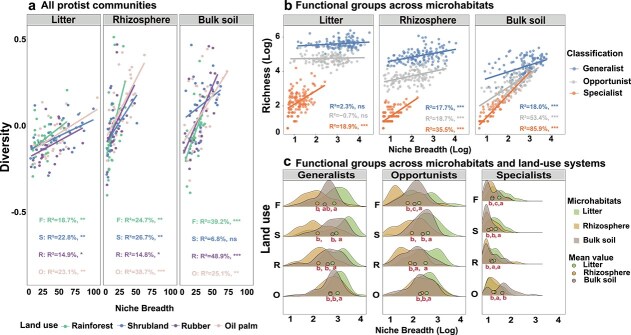
Relationships between niche breadth, diversity, and ecological strategy of protist communities across microhabitats and land-use systems. (**a**) Niche breadth versus diversity (Shannon residuals) in litter, rhizosphere, and bulk soil. Colours represent land-use systems; lines represent linear fit with *R*^2^ and significance. (**b**) Log-transformed ASV richness versus niche breadth for generalists, opportunists, and specialists. (**c**) Niche breadth distributions by strategy, microhabitat, and land use. Curves represent microhabitats; circles represent group means; letters represent significant differences (*P* < .05). Significance: ^*^P < .05, ^**^P < .01, ^***^P < .001; ns means not significant.

Overall, protist niche breadth was positively correlated with diversity, though the strength varied by microhabitat and land use. In litter, correlations were significant across all systems, while in rhizosphere and bulk soil they were strongest in rubber and oil palm, respectively (Fig. 4a).

Niche breadth significantly predicted protist richness across functional groups, with generalists showing the highest richness, followed by opportunists and specialists (Fig. 4b). The niche–richness relationship was strongest in specialists, particularly in bulk soil (*R*^2^ = 85.9%), weaker in generalists and intermediate in opportunists.

Further, niche breadth varied significantly with land-use system, protist functional group, and microhabitat, as indicated by a three-way interaction (*F*_4,1048_ = 4.57, *P* = .001). Generalists consistently exhibited the broadest niche breadth, particularly in the litter of rainforest and shrubland, with differences diminishing in rhizosphere and bulk soil, and being absent in oil palm plantations (Fig. 4c). Specialists showed the narrowest niche breadth, especially in oil palm plantations. Opportunists generally displayed intermediate niche breadth.

## Discussion

We investigated soil protist communities across litter, rhizosphere, and bulk soil, and their responses to land-use change. Contrary to our first hypothesis, rhizosphere and bulk soil communities responded more strongly than litter. Supporting our second hypothesis, litter was mainly shaped by abiotic factors, rhizosphere by biotic interactions, and bulk soil by both factors. In line with our third hypothesis, generalists maintained broad niches and stable richness, specialists showed niche contraction and richness loss and opportunists displayed intermediate responses. Overall, land-use change restructures protist communities in a microhabitat-specific manner, altering niche dynamics and influencing community assembly and ecosystem functioning. Separately analysing litter, rhizosphere, and bulk soil, the results revealed contrasting diversity and homogenization patterns that remain undetected if only bulk soil is analysed.

### Microhabitat filtering as dominant driver of protist communities

Microhabitat filtering—where specific conditions favour or constrain certain microbial taxa—has emerged as a dominant driver of soil community assembly [17, 75]. Our α- and β-diversity analyses showed that microhabitat was the main driver of protist diversity and composition, especially under land-use intensification in the rhizosphere. Consistently low diversity in the rhizosphere suggests strong filtering by roots, likely via exudation and competition favouring fast-growing or root-associated taxa [17, 76]. This supports our second hypothesis, as rhizosphere protist communities were primarily shaped by biotic factors such as bacterial, fungal, and nematode diversity. Interestingly, contrary to expectations of biodiversity loss under land-use intensification, we observed that protist α-diversity increased in intensively managed systems, especially oil palm plantations in the rhizosphere. These results support previous observations that agricultural intensification increases microbial richness but reduces community differentiation (β-diversity), resulting in more homogeneous communities [27, 77]. Protist communities in the rhizosphere and bulk soil responded more strongly to land-use intensification than those in litter, with the most pronounced shifts in oil palm, suggesting greater ecological sensitivity or stronger environmental filtering in rhizosphere and bulk soil [75, 78].

Differences in resource availability and microbial interactions likely contributed to the observed variations in protist community structure across microhabitats. In oil palm plantations, the rhizosphere showed increased α-diversity and strong compositional shifts, consistent with evidence that root-derived labile carbon enhances microbial activity and trophic complexity [79]. In oil palm plantations, palm fronds are cut and piled up in rows, thereby reducing the input of litter to the soil and shifting resource inputs towards roots and rhizosphere microorganisms [20, 80]. The SEM indicated that trophic interactions structure protist diversity in the rhizosphere, with α-diversity driven by bacterial, fungal, and nematode diversity, and β-diversity weakly associated with bacterial and fungal communities. In contrast, bulk soil was governed mainly by abiotic factors, with pH as the dominant filter, likely influenced by liming and fertilization. Changes in pH alter microbial activity and nutrient availability, indirectly shaping protist communities, consistent with studies reporting pH-driven shifts across microbial groups [16, 31, 81]. Litter protist communities showed stable α-diversity across land uses but elevated β-diversity in rainforest and shrubland, suggesting turnover driven by habitat filtering of specialists. Weaker niche constraints and variable litter inputs further indicate stronger stochastic structuring in litter [82, 83].

### Land-use intensification promotes protist community homogenization

Our β-diversity analyses revealed community homogenization, especially in the rhizosphere of oil palm plantations. In contrast, β-diversity in litter remained high, driven by specialist taxa, while richness and niche breadth of generalists changed little with land-use intensity. This indicates that litter communities are mainly structured by stochastic processes, whereas bulk soil and rhizosphere are more strongly shaped by deterministic filtering under intensive land use. These results align with studies showing that intensification reduces β-diversity across microbial, plant, and animal communities [7, 10, 84], largely through generalist expansion and specialist decline [27, 77].

The homogenization of protist communities under intensive land use likely resulted from the combined effects of abiotic and biotic factors. Agricultural practices, such as fertilization, liming, and monoculture plantations, create uniform soil physicochemical conditions, and this results in simplified trophic networks and reduced ecological niches. Further, frequent disturbances, e.g. due to more intensive exposure to climatic variations in plantations with more open canopies, likely contribute to more homogenous protist communities in plantations [10]. Beyond abiotic filtering, our SEM results highlight the important role of biotic interactions in shaping protist community structure. These findings emphasize that biotic filtering and cross-kingdom interactions are key drivers of protist homogenization. Notably, biotic homogenization resulting from intensive land use likely simplifies microbial food webs, and this may compromise nutrient cycling and, consequently, ecosystem resilience [4, 27]. Supporting this conclusion, reduced β-diversity and the loss of specialized taxa critical for multiple ecosystem processes are linked to diminished soil functioning [85]. Furthermore, dominance of generalist protists with high niche overlap and functional redundancy may suppress trophic interactions and compromise long-term soil stability [86].

### Niche dynamics of protist communities across microhabitats

Supporting our third hypothesis, litter-associated protists showed broader ecological niches, whereas bulk soil protists were more specialized. This pattern aligns with ecological theory predicting that variable environments, such as in the litter, favour generalists, while stable, resource-partitioned habitats like bulk soil promote specialization [33, 37]. The narrowing of niche breadth among specialist taxa in the rhizosphere and bulk soil supports the concept of niche compression, where environmental filtering and habitat homogenization constrain the range of conditions species can occupy [87, 88].

Litter, dominated by generalist protists, is exposed to variable environments that select for broad tolerance and resource use [4, 27, 37], explaining the relatively stable diversity across land-use systems. By contrast, rhizosphere and bulk soil communities show greater niche specialization, likely driven by biotic interactions and resource partitioning [89]. Intensive practices, especially in oil palm, may intensify these pressures and reduce β-diversity. These patterns underscore the role of niche-based traits in community stability. Opportunists displayed intermediate niche breadth and inconsistent responses, reflecting sensitivity to short-term resource shifts. While generalists buffer communities against disturbance, the loss of specialists reduces trophic complexity and complementarity, promoting redundancy and undermining stability [85, 86].

In conclusion, land-use intensification reshapes soil protist communities in a microhabitat-specific manner. Litter communities remain stable, while rhizosphere and bulk soil shift markedly under the combined influence of biotic and abiotic factors. These changes favour generalists with broad tolerance but reduce functional specialists, especially in oil palm plantations. Consequently, shifts in rhizosphere and bulk soil cause functional homogenization, reduced β-diversity, and threat to the stability of soil food web resilience. Protists thus serve as sensitive bioindicators of soil degradation. To sustain biodiversity and ecosystem functions, land-use practices should preserve microhabitat structure and support trophic complexity.

## Supplementary Material

Supplementary_Figures_update_ycag132

Supplementary_Tables_final_ycag132

## Data Availability

The 18S rRNA gene sequences were deposited in the National Center for Biotechnology Information (NCBI) Sequence Read Archive (SRA) under the study accession number PRJNA1266886.
