# Supplementary material for: Microhabitat-specific restructuring of soil protist communities along a tropical land-use intensification gradient
Source: ISME Commun. 2026 May 15;6(1):ycag132. doi: 10.1093/ismeco/ycag132 (PMC13271387; doi:10.1093/ismeco/ycag132)
Supplement: Supplementary_Figures_update_ycag132 [file supplementary_figures_update_ycag132.docx]

**Microhabitat-specific restructuring of soil protist communities along a tropical land-use intensification gradient**

Gennuo Wang, Yan Zhang, Zheng Zhou, Carina C.M Moura, Zuopeng Liu, Xu Xu, Siti Meliah, Christian Stiegler, [Fabian Brambach](http://www.uni-goettingen.de/de/587688.html), Martyna M. Kotowska, Rahayu Widyastuti, Ingo Grass, Catrin Westphal, Stefan Scheu, and Valentyna Krashevska

**Supplementary Figures**

**
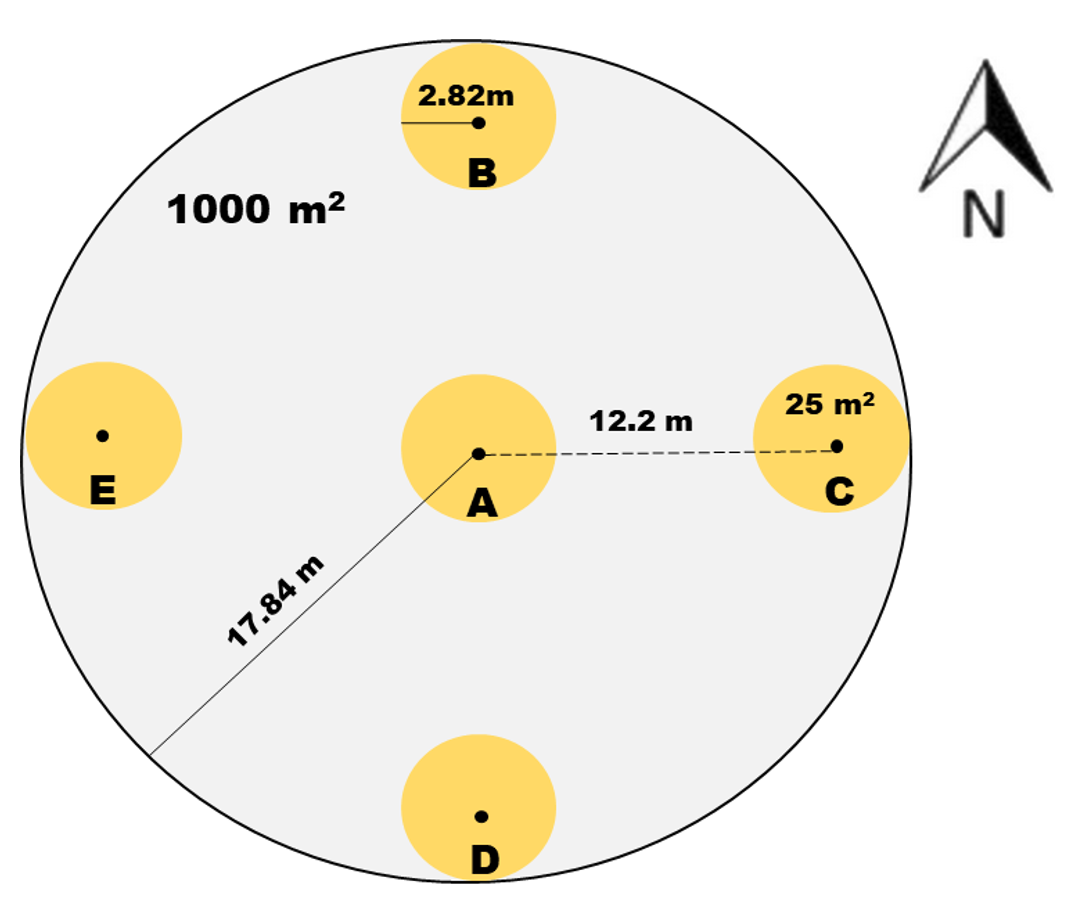
**

**Supplementary Fig. 1. Study plot with five sub-plots (A-E).** The plot has a total area of 1000 m^2^. Subplot A is central; B–E are placed in cardinal directions at 12.2 m distance. Locations avoid edge effects (>50 m homogeneous land use). In oil palm, subplot A is between rows; B and C are placed in the palm circle and frond stack.

**
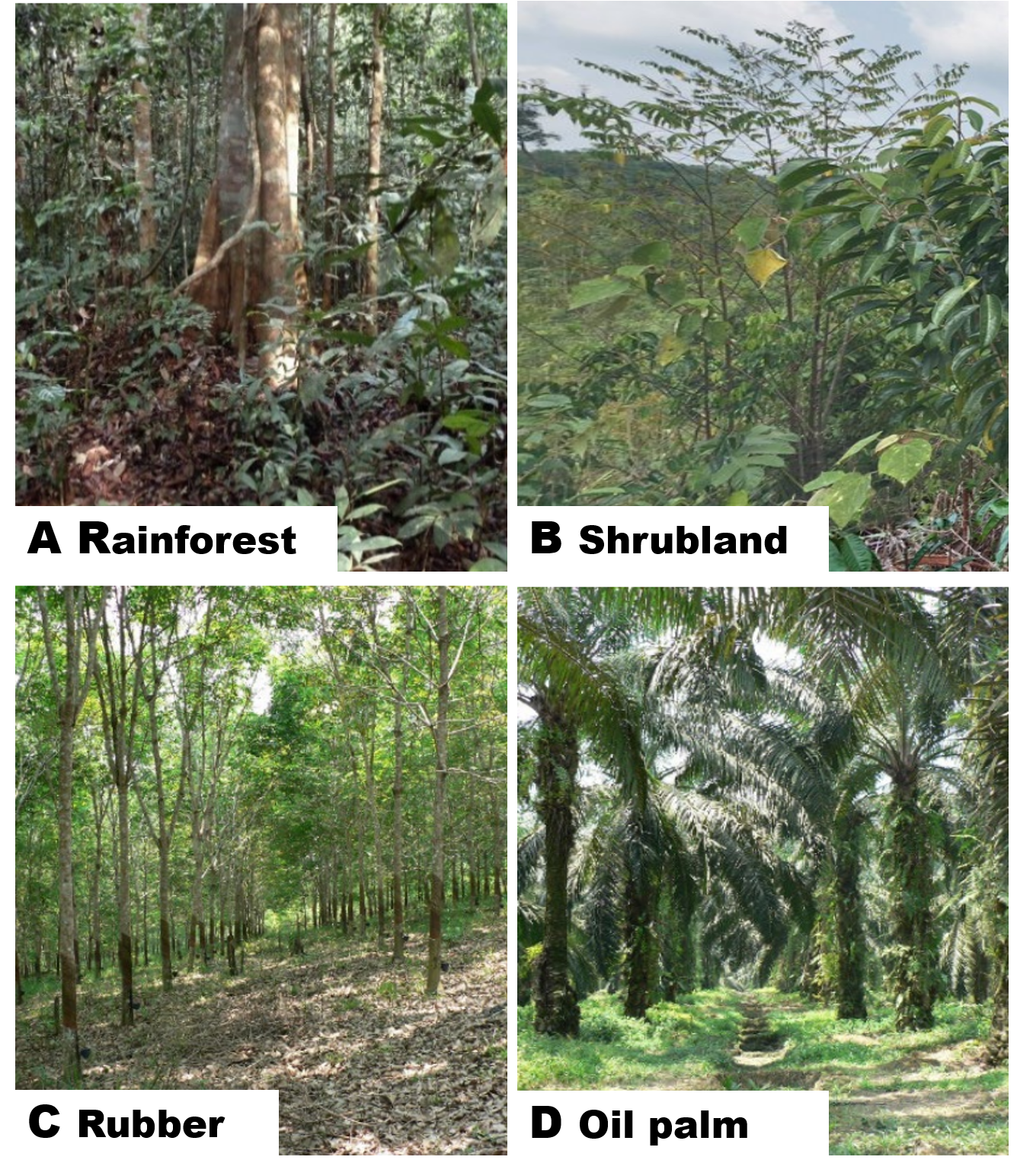
**

**Supplementary Fig. 2. Study sites on the different land-use systems:** rainforest (**A**), shrubland (**B**), rubber plantations (**C**) and oil palm plantations (**D**)**.** Samples were collected from three microhabitats per plot: litter (n = 124), rhizosphere (n = 125) and bulk soil (n = 123). All photos were obtained from the CRC990/EFForTS collaborative research project website (https://www.uni-goettingen.de/de/310995.html).


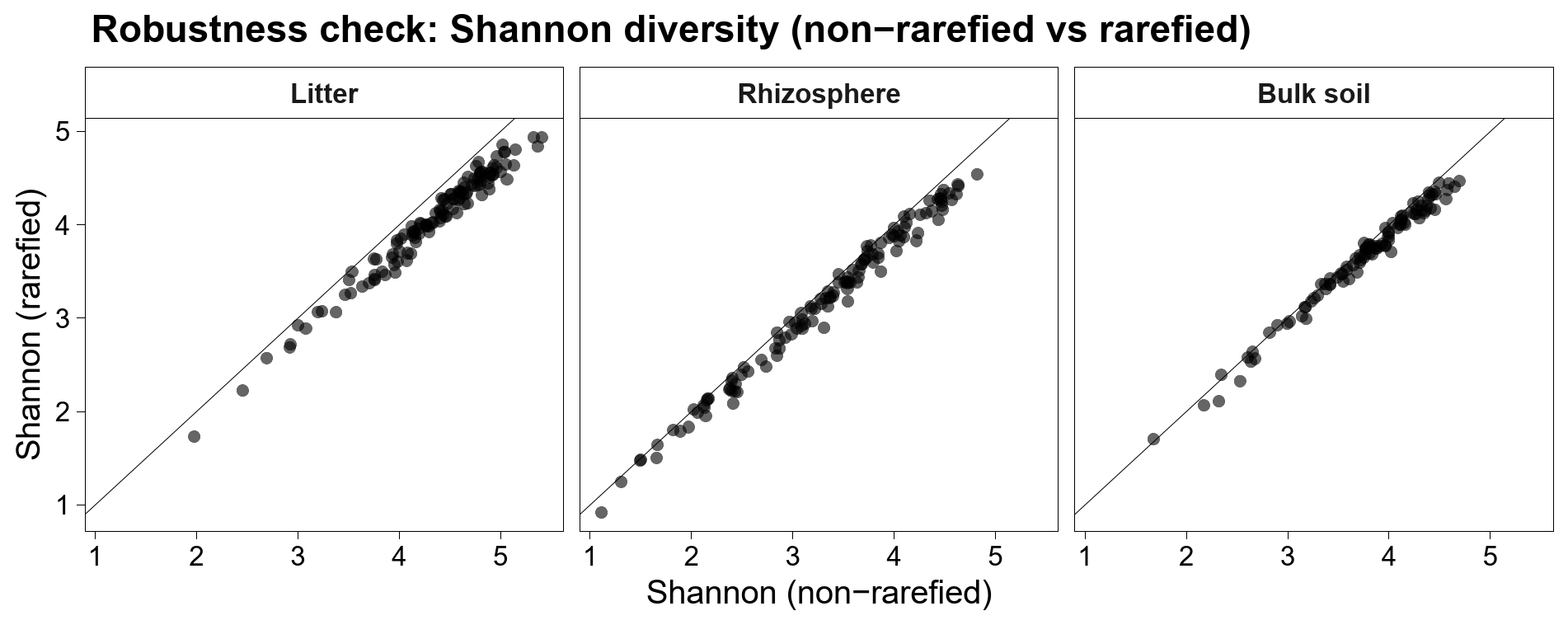


**Supplementary Fig. 3. Robustness check comparing non-rarefied and rarefied Shannon diversity across microhabitats.** Each point represents one sample. Panels show litter, rhizosphere, and bulk soil, respectively. The solid line indicates the 1:1 relationship.





**Supplementary Fig 4. Correlation structure and multicollinearity assessment of environmental and biological variables across microhabitats.** (A–C) Pairwise Pearson correlation matrices for litter (**A**), rhizosphere (**B**) and bulk soil (**C**). Circle size and color represent the strength and direction of Pearson’s correlation coefficients (r), with blue indicating positive and red indicating negative correlations. (D–F) Variance inflation factors (VIF) for variables in the corresponding microhabitats (litter - **D**, rhizosphere - **E**, bulk soil - **F**). The dashed red line indicates the threshold (VIF = 5), above which multicollinearity may distort model performance.


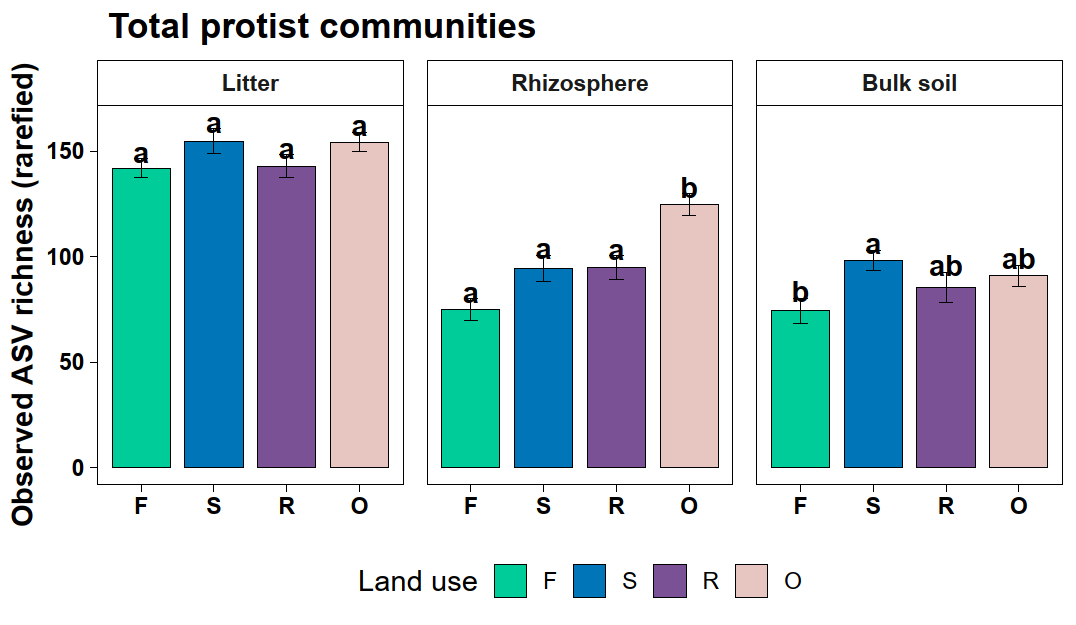


**Supplementary Fig 5.** Protist richness in litter, rhizosphere, and bulk soil under four land-use systems (F = rainforest, S = shrubland, R = rubber plantations, O = oil palm plantations). Bars represent mean ± SE; different letters indicate significant differences (ANOVA, Tukey’s HSD, *P* < 0.05).


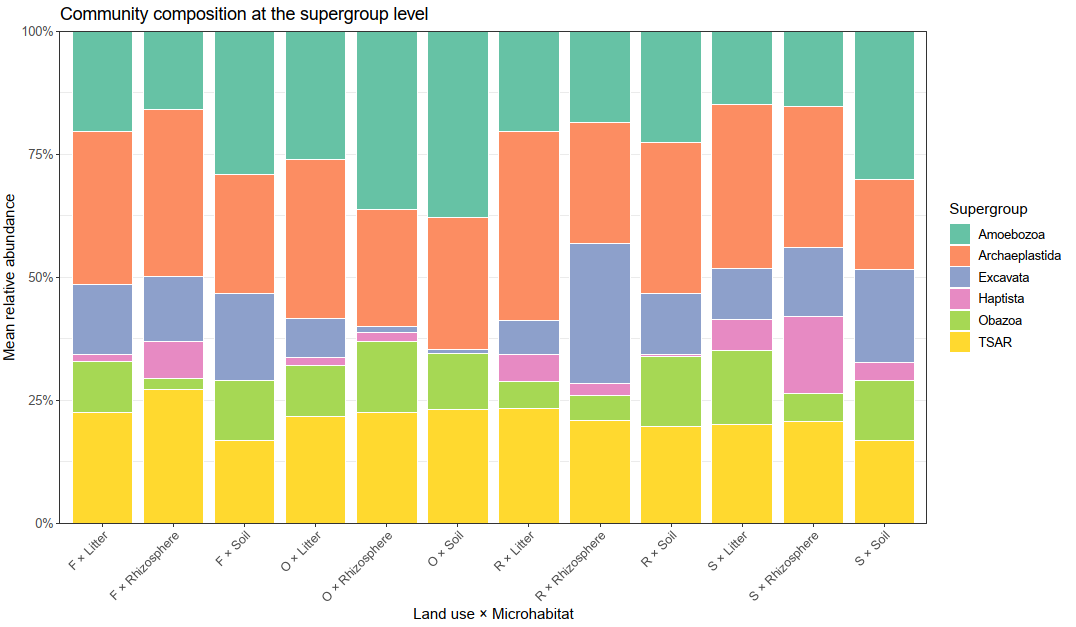


**Supplementary Fig 6**. **Community composition of protists at the supergroup level across land-use systems and microhabitats**. Stacked bar plots show the mean relative abundance (%) of major protist supergroups. Bars represent combinations of land-use systems (F, rainforest; S, shrubland; R, rubber plantation; O, oil palm plantation) and microhabitats (litter, rhizosphere, and bulk soil). Relative abundances were calculated based on ASV read proportions and averaged across samples within each land use × microhabitat combination.


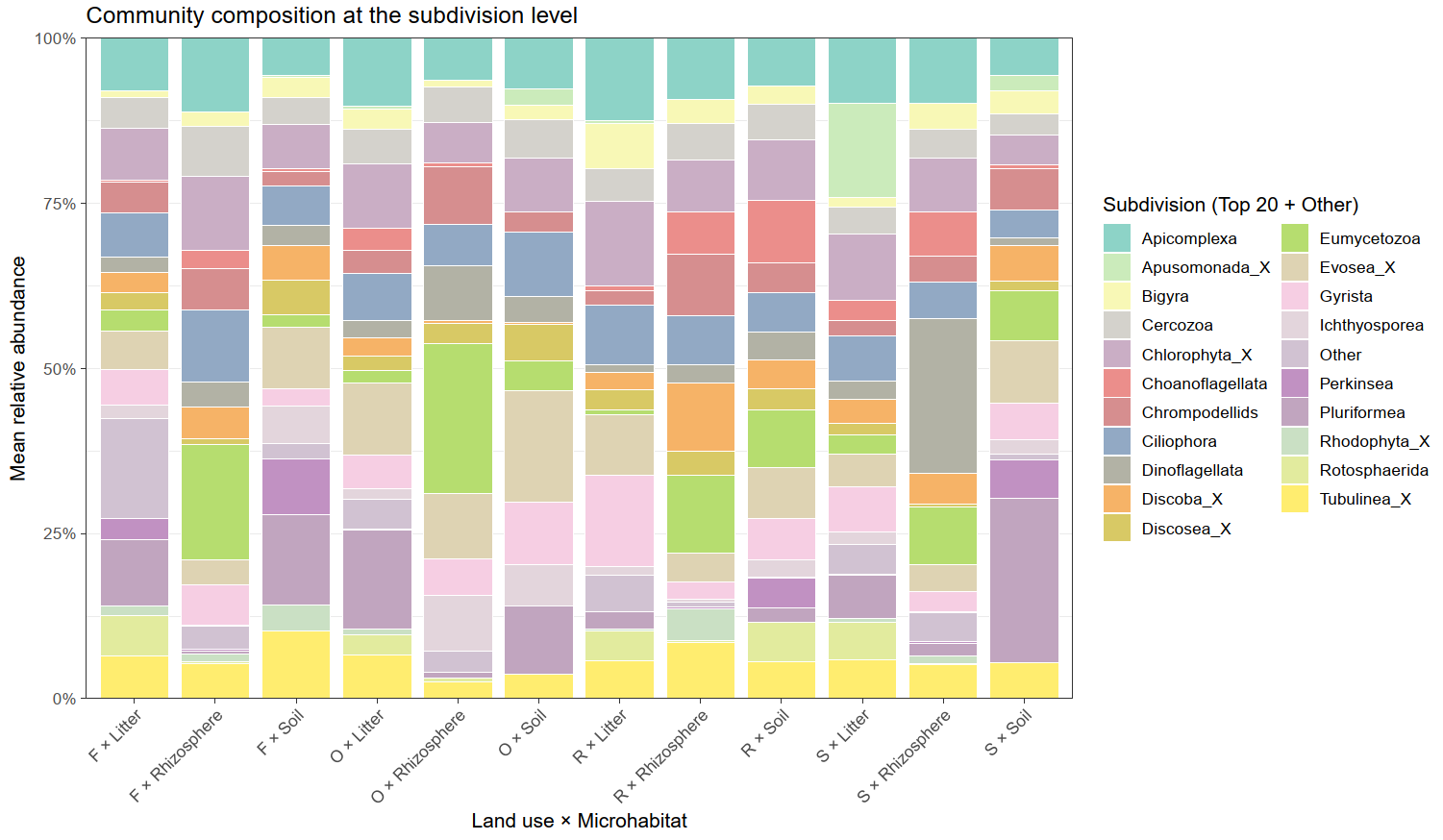


**Supplementary Fig 7**. **Community composition of protists at the subdivision level across land-use systems and microhabitats**. Stacked bar plots show the mean relative abundance (%) of the 20 most abundant protist subdivisions, with all remaining taxa grouped as “Other”. Bars represent combinations of land-use systems (F, rainforest; S, shrubland; R, rubber plantation; O, oil palm plantation) and microhabitats (litter, rhizosphere, and bulk soil). Relative abundances were calculated based on ASV read proportions and averaged across samples within each land use × microhabitat combination.


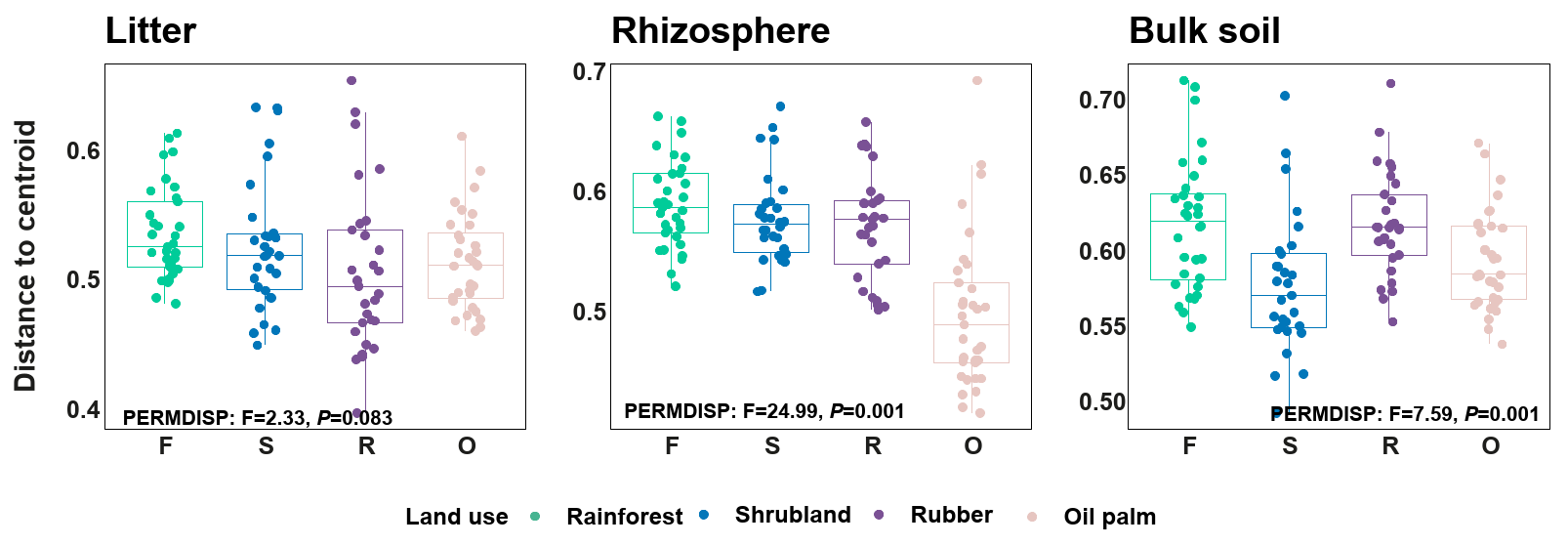


**Supplementary Fig 8. Multivariate dispersion of protist communities in litter, rhizosphere, and bulk soil across four land-use systems**. Dispersion was quantified as the distance to group centroid based on community dissimilarities. Land-use systems include rainforest (F), shrubland (S), rubber plantations (R), and oil palm plantations (O). Boxes represent interquartile ranges with median values; points represent individual samples. PERMDISP results (F and *P* values) are shown in each panel.


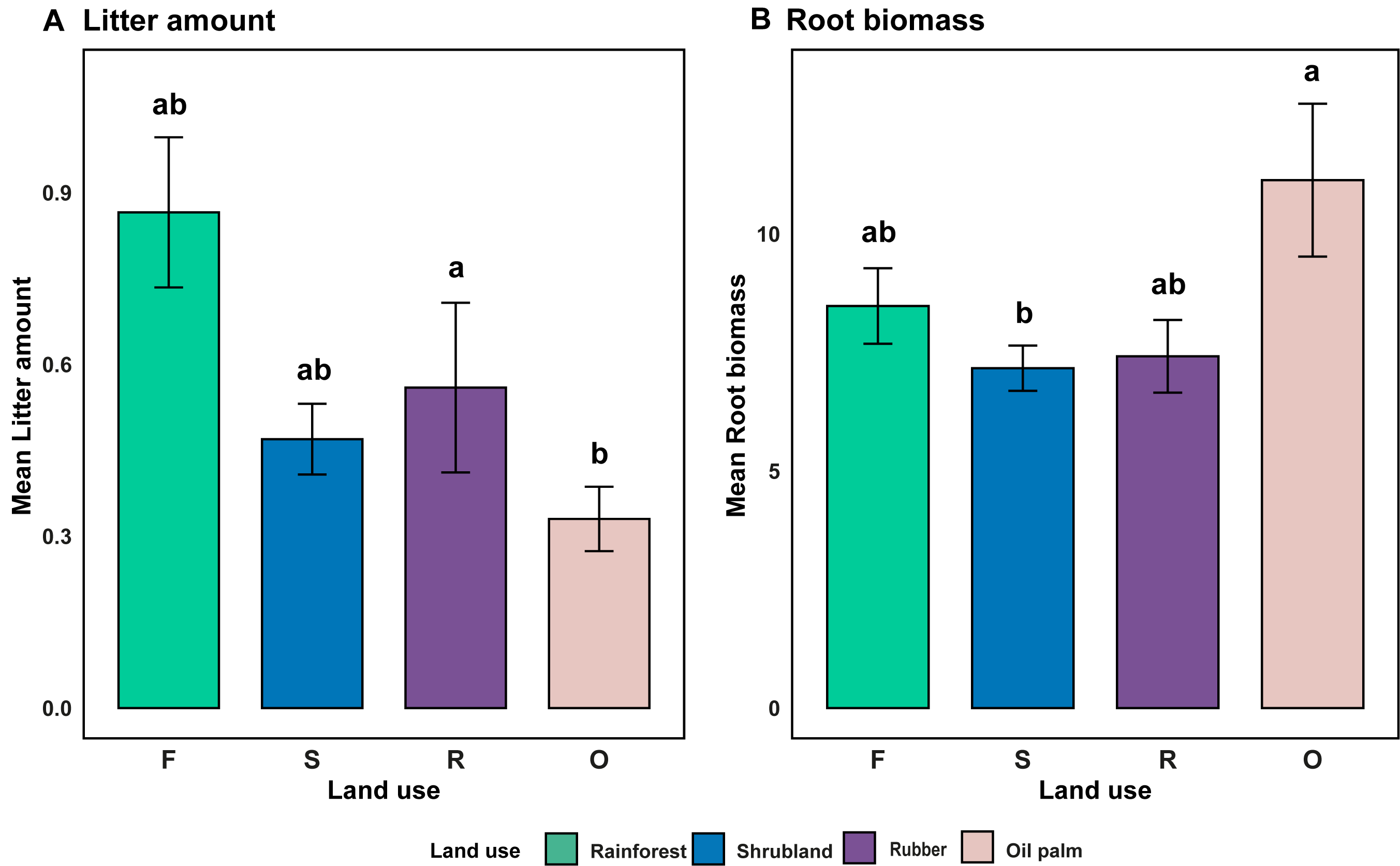


**Supplementary Fig 9**. **Mean litter amount (A) and root biomass (B) in rainforest, shrubland, rubber and oil palm plantations**. Data were obtained from field plots (N = 126 per land-use type), with values representing means ± standard error. Different letters above bars indicate significant differences among land-use types based on Tukey’s HSD test (*P* < 0.05).
